# Non-receptor tyrosine kinase Src is required for ischemia-stimulated neuronal cell proliferation via Raf/ERK/CREB activation in the dentate gyrus

**DOI:** 10.1186/1471-2202-10-139

**Published:** 2009-11-27

**Authors:** He-Ping Tian, Bao-Sheng Huang, Jie Zhao, Xiao-Han Hu, Jun Guo, Li-Xin Li

**Affiliations:** 1 Department of Neurosurgery, First Affiliated Hospital of Nanjing Medical University, Nanjing 210029, China; 2 Jiangsu Provincial Key Laboratory of Human Functional Genomics, Nanjing Medical University, Nanjing 210029, China; 3 Department of Biochemistry and Molecular Biology, Nanjing Medical University, Nanjing 210029, China

## Abstract

**Background:**

Neurogenesis in the adult mammalian hippocampus may contribute to repairing the brain after injury. However, Molecular mechanisms that regulate neuronal cell proliferation in the dentate gyrus (DG) following ischemic stroke insult are poorly understood. This study was designed to investigate the potential regulatory capacity of non-receptor tyrosine kinase Src on ischemia-stimulated cell proliferation in the adult DG and its underlying mechanism.

**Results:**

Src kinase activated continuously in the DG 24 h and 72 h after transient global ischemia, while SU6656, the Src kinase inhibitor significantly decreased the number of bromodeoxyuridine (BrdU) labeling-positive cells of rats 7 days after cerebral ischemia in the DG, as well as down-regulated Raf phosphorylation at Tyr(340/341) site, and its down-stream signaling molecules ERK and CREB expression followed by 24 h and 72 h of reperfusion, suggesting a role of Src kinase as an enhancer on neuronal cell proliferation in the DG via modifying the Raf/ERK/CREB cascade. This hypothesis is supported by further findings that U0126, the ERK inhibitor, induced a reduction of adult hippocampal progenitor cells in DG after cerebral ischemia and down-regulated phospho-ERK and phospho-CREB expression, but no effect was detected on the activities of Src and Raf.

**Conclusion:**

Src kinase increase numbers of newborn neuronal cells in the DG via the activation of Raf/ERK/CREB signaling cascade after cerebral ischemia.

## Background

It is well established that the generation of new neurons continues throughout adulthood in the DG in many species of vertebrates [1,2]. The neuronal cell proliferation in the DG is regulated by several physiological factors, including enriched environments [3], running [4] and learning [5]. Reportedly, such pathological states as lesion and ischemia may result in cell birth in the hippocampal DG [6,7]. Elegant studies in multiple labs have described the maturation of newborn cells in the subgranular region of DG and their eventual incorporation into mature physiologically active dentate granule neurons [8,9]. After transient forebrain ischemia, newly generated neurons migrate and incorporate into functional synaptic circuitry, which provides a possible therapeutic strategy for ischemic injury repair [10]. The identification of intracellular signaling events that regulate the rate of ischemia-induced progenitor cell proliferation is therefore of significant interest.

The Src family kinases (SFKs) are a family of proteins that have been implicated in relaying signals as downstream of a wide variety of cell-surface receptors to regulate diverse cellular responses including proliferation, differentiation, survival changes in cellular architecture, and regulating cell adhesion and migration [11]. Five members of SFKs (Src, Fyn, Yes, Lck, and Lyn) are known to be expressed in the mammalian brain [12], of which Src, Fyn, and Yes have been detected in the developing brain [13]. Furthermore, differentiating rodent neurons express a high level of Src, which is identified as being important in growth cone-mediated neurite extension, synaptic plasticity, and neuronal differentiation [14,15]. The increment in Src kinase activity observed during the development of striatum and hippocampus is coincident with the peak period of neurogenesis and neuronal growth [16]. Nevertheless, its association with ischemia-induced neuronal cell proliferation in the hippocampal DG and potential signal transduction has not been explored.

One signaling cascade implicated in regulating the proliferative capacity of adult stem cells is mitogen-activated protein kinase (MAPK) [17]. The extracellular signal-regulated kinase (ERK) is a subfamily member of MAPK activated by an upstream kinase called MAPKKK (Raf)/ERK kinase (MEK) in response to growth stimuli [18]. Much evidence exists that the ERK pathway plays a role in progenitor cell proliferation or differentiation in a number of model systems mediated by alterations of nuclear transcription factors [17]. For example, the ERK pathway is involved in neurogenesis, neurite outgrowth, and neuronal survival induced by neurotrophic factors [19,20] and pharmacon like valproate [21] or lithium [22]. Meanwhile, MAPKKK (Raf) is reported to be potently phosphorylated by Src kinase at Tyr340/341 residue in mammalian cells, relieving its autoinhibition [23]. No data to date is available regarding the question whether ERK triggers cell proliferation after ischemia in the DG region of hippocampus as well as the role of Src kinase in the process.

Non-receptor tyrosine kinase Src plays prominent roles in ischemic cerebral apoplexy [24]. Ischemic challenge particularly results in a sustained activation of the Src family PTKs (primarily Src) in the rat hippocampus [25,26]. In this study, we propose that sustained activation of Src kinases plays a key role in progenitor cell proliferation via Raf/ERK/CREB cascades in hippocampal dentate gyrus after transient cerebral ischemia.

## Results

### Src kinase was sustainedly activated in the hippocampal DG following ischemia-reperfusion and its inhibitor SU6656 prevented ischemia-induced neuronal cell proliferation

**Figure 1:**
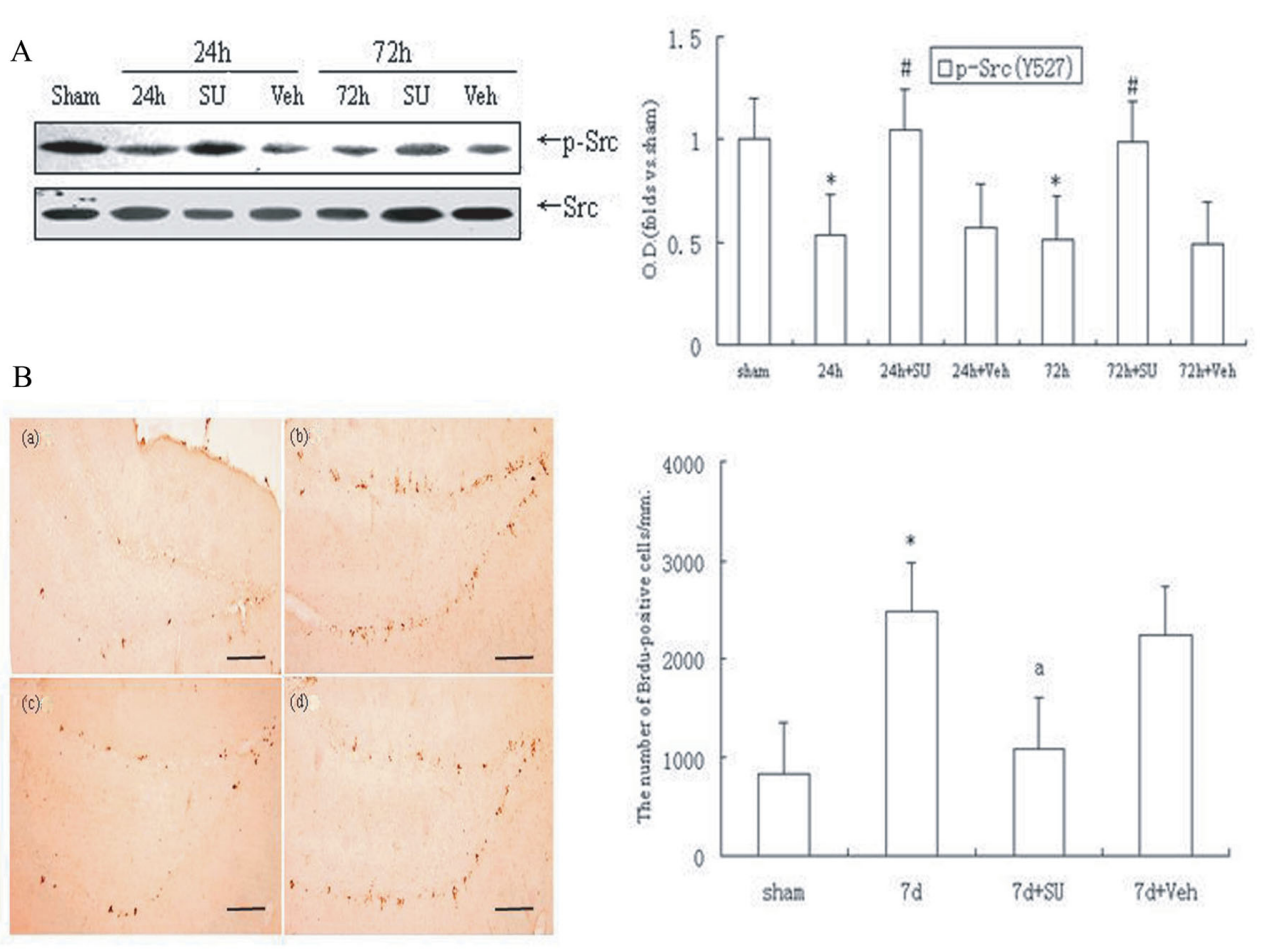
**Src kinase sustainedly activated in the DG field of hippocampus after ischemia, while its inhibitor SU6656 blocked cell proliferation induced by ischemia**. SU6656 was intracerebroventricularly (i.c.v) administratered 20 min before ischemia. (A) Western blot for Src kinase detected by anti-p-Src antibody (Tyr527). (B) DAB staining for Brdu-positive cells in the SGZ of dentate gyrus. (a) Sham rats (n = 4), (b) Rats with 7 days of reperfusion postischemia (n = 5), (c) SU6656-treated ischemic rats (n = 5), and (d) vehicle-treated ischemic rats (n = 4). *P < 0.05 vs. sham group; ^#^*P *< 0.05 or ^a^*P *< 0.05 vs. the respective reperfusion group. Bar: 200 μm.

Our previous studies indicated that cerebral ischemia induced sustained activation of Src kinase following ischemia in the hippocampus [25]. In the present study, we further assessed the activity of Src kinase of DG/CA3 subfield after 24 h reperfusion post-ischemia. Based on that the phosphorylation of Src C-terminal Tyr-527 residue expresses a down-regulated kinase state, anti-Src Tyr527 antibody was employed, as exhibited in Fig. 1A (*P *< 0.05), the activity of Src remained in a high level until 24 h and 72 h reperfusion illustrated by strong dephosphorylation of Src at Tyr527 site compared with sham operated animals. It suggested that continuous Src kinase activation may be involved in triggering some pathological phenomena in the DG/CA3 region induced by ischemia and reperfusion. As has been well accepted, ischemia stimulates neurogenesis in the DG; thus we explored the possibility of Src being involved in the proliferation of adult hippocampal progenitor cells. SU6656, an inhibitor of Src kinases, was administered into cerebral ventricle before ischemia, and found to be effective in suppressing Src activity (*P *< 0.05, Fig. 1A). On day 7 post ischemia, the number of BrdU-positive cells was shown to be approximately 4-fold larger in the ischemic group than that in the sham group. BrdU-labeled cells in each group were located exclusively in the SGZ in clusters (*P *< 0.05, Fig. 1B - a, b). Importantly, we demonstrated that SU6656 decreased the number of BrdU - positive cells after ischemia (P < 0.05, Fig. 1B - c). The solvent had no influence on the number of BrdU-positive cells on the 7^th ^post-ischemic day as opposed to the ischemic group (Fig. 1B - d). Based on the results, we surmise that Src kinases may be implicated in the ischemia-induced cell proliferation in the hippocampal DG.

### SU6656 inhibit Raf (340/341)/ERK/CREB cascade in the DG after ischemia

**Figure 2:**
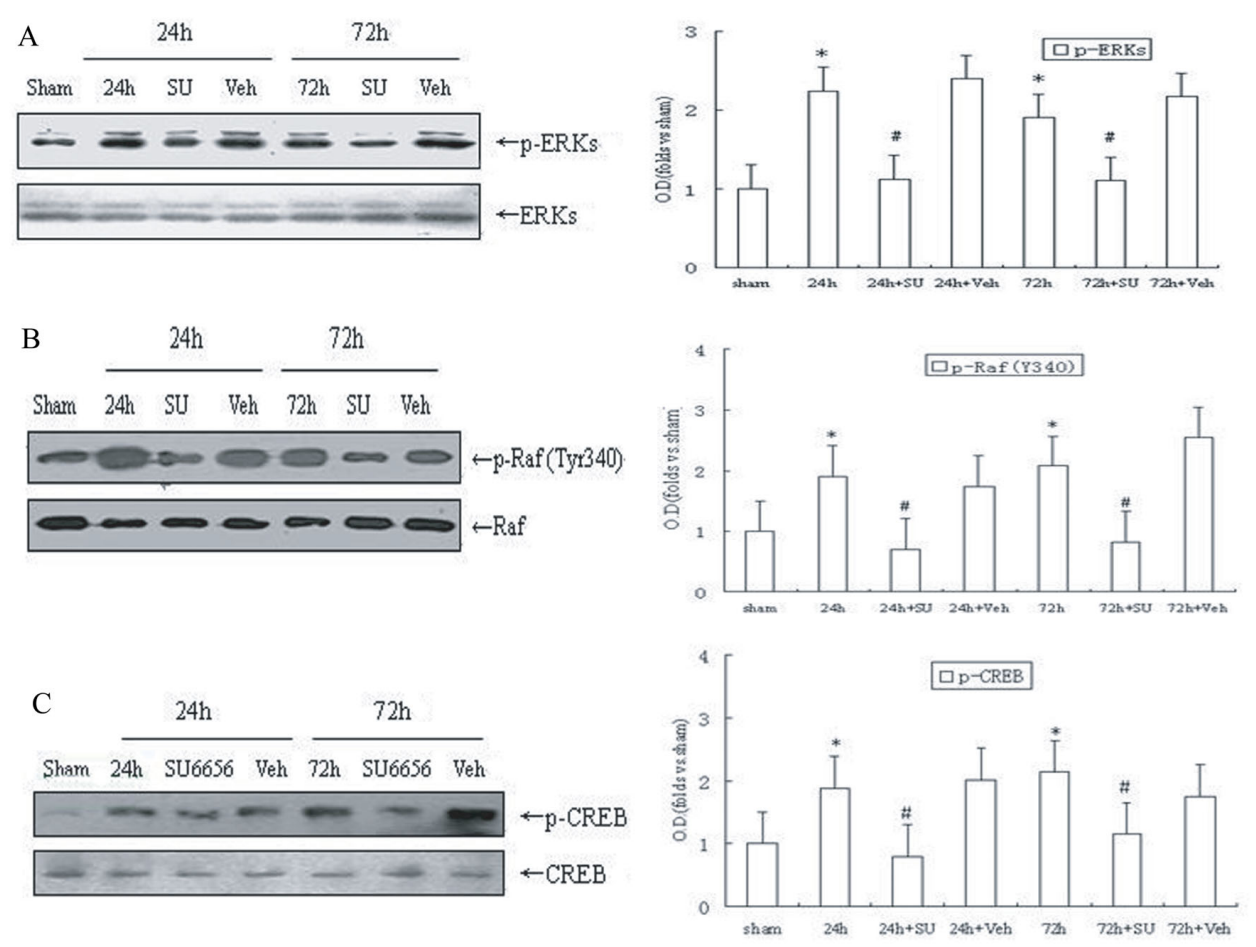
**Inhibiting the activation of Src kinase by SU6656 down-regulate Raf (Y340)/ERK/CREB cascade after ischemia- reperfusion**. Samples were used as in Fig. 1. (A) Raf phosphorylation at Tyr340/341. (B) ERK phosphorylation at Thr202/Tyr204. (C) CREB phosphorylation at Ser133. Data are expressed as mean ± S.D. (n = 4/group) *P < 0.05 vs. sham group, ^#^*P *< 0.05 vs. respective reperfusion group.

For a better understanding of the down-stream signaling mechanism of Src on cell proliferation stimulated by ischemia, some signaling proteins relating to growth like ERK or CREB were examined in the following way. First, we attempted to see whether the alteration of Src kinases affected the ischemia-induced ERK activity in the areas of CA3 and DG. We selected two time spots (24 h and 72 h). At both spots the amount of p-ERK increased at least two-fold when compared with sham control group; while the elevated level of p-ERK lowered in the SU6656 - treated rats (*P *< 0.05, Fig. 2A), and the solvent group showed no change in the phosphorylation of ERK after ischemia and reperfusion(*P *> 0.05, Fig. 2A). These results suggest that ERK phosphorylation in the DG region triggered by ischemia depends on Src activation. To further explore how Src kinase induced ERK activation, we examined the effects of SU6656 on Raf activity in CA3 and DG fields following ischemia. Raf, an up-stream kinase of ERK, is thought to be activated by its Tyr340/341 - phosphorylation. As demonstrated in (*P *< 0.05, Fig. 2B), drastic phosphorylation of Raf at these residues was observed after 24 h and 72 h reperfusion. SU6656, rather than the solvent, markedly attenuated the effect (*P *< 0.05, Fig. 2B), indicating that Src kinase may trigger the activation of ERK via phosphorylation of Raf at its Tyr340/341 residues after ischemia and reperfusion. Besides, another protein CREB, a transcription factor involved in the cell proliferation in various models, was assessed. The phosphorylated level of CREB (Ser133) grew after the 24 h and 72 h reperfusions respectively, but strikingly down-regulated at both time spots in the group of SU6656. The vehicle-treated group kept unchanged (*P *< 0.05, Fig. 2C), suggesting a role of CREB in Src-dependent cell proliferation after ischemia. On the other hand, the presence of the SU6656 and its solvent did not alter the level of total ERK, Raf and CREB proteins (Fig. 2A, B and 2C). Therefore, our study revealed the involvement of Src kinase in the regulation of Raf/ERK and CREB cascade in the DG fields after ischemia.

### U0126 block generation of new neurons in the DG through inhibition of ERK/CREB cascade after cerebral ischemia

**Figure 3:**
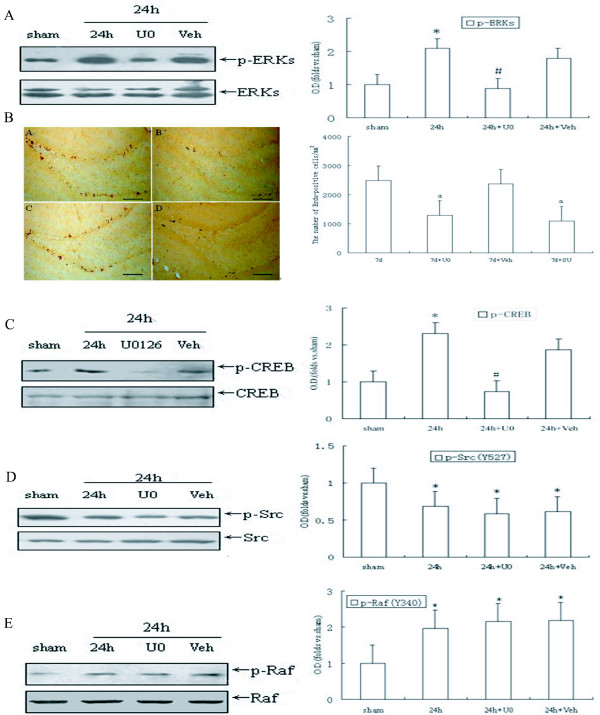
**Inhibition on activated ERK blocked ischemia-stimulated cell proliferation in the dentate gyrus and CREB activation, but it did not affect expression of p-Src and p-Raf**. U0126 was intracerebroventricularly (i.c.v) administratered 20 min before ischemia. (A) ERK phosphorylation after reperfusion 24 h post ischemia.(B) DAB staining for BrdU-positive cells in the SGZ at the 7^th ^post-ischemic day. (a) Rats with 7 days of reperfusion after ischemia (n = 5), (b) U0126-treated ischemic rats (n = 4), (c) vehicle-treated ischemic rats (n = 4), and (d) SU6656-treated ischemic rats (n = 5). (C) CREB phosphorylation, (D) Src phosphorylation or (E) Raf phosphorylation. Data are expressed as mean ± S.D. *P < 0.05 vs. sham, ^#^*P *< 0.05 or ^a^*P *< 0.05 vs. respective reperfusion. Bar: 200 μm

To determine whether ERK pathway participate in cell proliferation of DG induced by ischemia, we used U0126 as its inhibitor after being infused into bilateral cerebral ventricle, and it turned out to be effective in depressing ERK activity (*P *< 0.05, Fig. 3A). Consistent with our expectation, we demonstrated that U0126 had a similar effect on SU6656, which significantly decreased the number of BrdU-labeled cells in the SGZ of DG field 7 days after ischemia (*P *< 0.05, Fig. 3B - a, b and 3Bd). The solvent of the U0126 group did not change the number of new born neurons following ischemia-reperfusion. These results indicate that SU6656 inhibited cell proliferation through down-regulated phosphorylation of ERK in the DG field. Subsequently, we observed the effects of U0126 on CREB activation after ischemia-reperfusion in the fields of CA3 and DG. The data showed that rats treated with U0126 before ischemia had lower phosphorylated level of CREB, in comparison with the 24 h reperfusion rats (*P *< 0.05, Fig. 3C), suggesting that CREB might be contributed to ERK dependent neural cell proliferation after ischemia. Activities of Src and Raf after U0126 treatment in the DG following ischemia showed that down-regulation of ERK had no relation to Src and Raf phosphorylation on these very residues at the time period of maximum stimulation of Src/Raf (*P *> 0.05, Fig. 3D and 3E), further proving that Src/Raf cascade was an upstream mediator for ERK activation. The solvent of U0126 exhibited no alterations on phosphorylation of ERK, Src, Raf and CREB after 24 h reperfusion, and no variation was detected in the total ERK, Src, Raf and CREB level in all the groups (Fig. 3A-E). The above results are suggestive of a key role of Src-stimulating Raf (Tyr340/341)/ERK/CREB pathway in the ischemia-induced hippocampal cell proliferation.

### Neurons of DG subfields are resistant to ischemia injury, and activation of Src but not ERK promote delayed neuronal death of CA1 region

**Figure 4:**
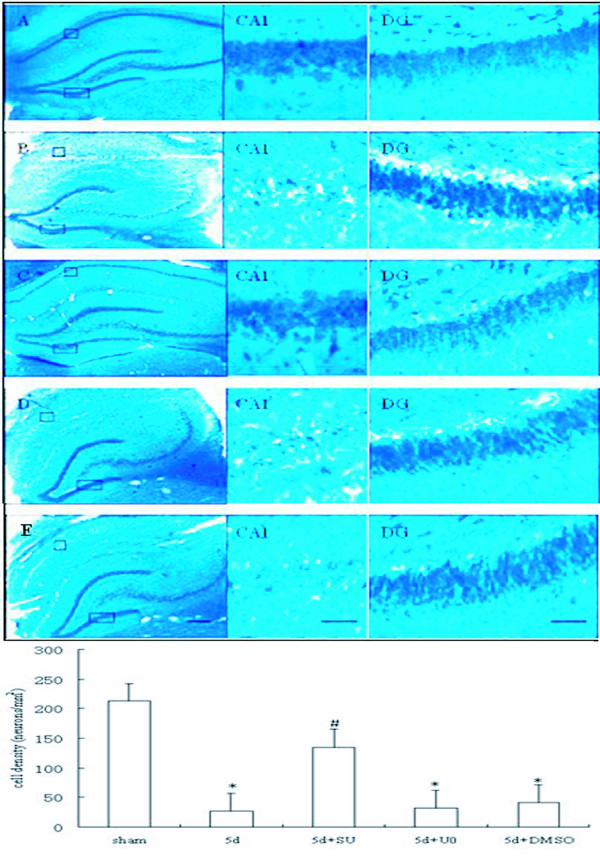
**The region of DG is resistant to ischemic damage while CA1 is vulnerable. Blockage activation of Src instead of ERK significantly decreased neuronal loss in hippocampal CA1 after ischemia**. NISSL staining was performed to examine the survival of hippocampal neurons following 5 days reperfusion. (A) sham group, (B) 5 days of reperfusion postischemia (R5d), (C) SU6656 administration, (D) U0126 administration, and (E) vehicle administration (n = 5). Data are expressed as means ± S.D. *P < 0.05 versus sham; ^#^*P *< 0.05 versus I/R 5 days groups (n = 5). Scale bars in A-E = 200 μm; bars in CA1 and DG = 20 μm.

Transient global cerebral ischemia leads to neuronal death of hippocampus. To investigate whether survival of hippocampal neurons was affected by SU6656 or U0126, NISSL staining was performed to detect hippocampal neurons in the rats subjected to 5 days of reperfusion following ischemia (I/R5d). Under a light microscope, the normal neurons showed round cell bodies and plain stained nuclei (Fig. 4A). After 5 days of reperfusion following brain ischemia, though the regions of CA3 and DG were shown to be the same as in the sham group and no damaged cell was detectable, a prominent neuronal loss was observed and the few pyramidal neurons left were shrunken with pyknotic nuclei in the hippocampal CA1 region (*P *< 0.05, Fig. 4B). However, administration with SU6656 before ischemia markedly increased the survival of neurons in the hippocampal CA1 region (*P *< 0.05, Fig. 4C - CA1), while infusion of U0126 (*P *> 0.05, Fig. 4D - CA1) or the solvent (*P *> 0.05, Fig. 4E - CA1) did not alleviate post-ischemic cell death. Statistical analysis of neuronal density for each group is shown in (Fig. 4). Besides increasing cell proliferation of DG, activation of Src also participated in pyramidal cell death in CA1 triggered by ischemia, but Src/Raf/ERK cascade only explained the new cell birth in the DG.

## Discussion

Cerebral ischemia insults have been shown to trigger proliferation of endogenous neural stem cells or progenitor cells located in the hippocampal dentate gyrus [27,28], and the number of neuronal stem cells increased obviously 5-7d after ischemia [22,28]. The pathological mechanism of ischemia is regulated by a variety of protein tyrosine kinases. Some of these kinases, including FGF, BDNF and NGF receptors, have been reported to be involved in ischemia-stimulated neurogenesis [29]. However, the roles of non receptor tyrosine kinases (mainly is Src) remain unclear. An earlier study reported that activation of Src kinase is related to hippocampal neurogenesis beginning in late embryonic life and continuing for some period of time after birth [16]. Recent studies indicate that Src activation is required for muscarinic receptor-mediated DNA synthesis and cell proliferation in neural precursor cells [30]. Interestingly, our present findings demonstrated that cerebral ischemia stimulated a sustained activation of Src kinase in the DG region, and SU6656, the Src specific inhibitor, significantly decreased the number of BrdU - positive cells of DG after 7 days of reperfusion (Fig. 1B). Thus, we suppose that Src activation following stroke insult is responsible for ischemia-induced cell proliferation in the dentate gyrus.

The molecular mechanism underlying Src kinase dependent neural cell proliferation stimulated by ischemia is complex. Signals that increase intracellular Ca^2+ ^concentration serve as a main contributing factor for activation of Src [31], which, as a cytoplasmic non receptor tyrosine kinase, acts like a relay station that transmits various signals to their specific targeted downstream molecules to modulate transcriptional activation and mitogenesis. Under some conditions, ERK has been demonstrated as one of the key downstream signals of Src kinase. The problem is whether ERK participates in the generation of new adult progenitor cells or not and whether its role is positive or negative, which awaits further exploration. Our previous studies showed that cerebral ischemia stimulated a sustained activation of ERK in the DG field of hippocampus [25,26], and in the present study the elevation was found lasting at least 72 h after ischemia (Fig. 2A), which is roughly coincident with activation of Src kinase (Fig.1A). Several studied have demonstrated that both ERK and Src immunoreactivity were enhanced in the DG after ischemic insult [32-34], suggesting that there may exists a relationship between the two. This presumption is supported by the detection of Raf, the well accepted ERK cascades upstream kinase, whose residues Tyr 340/341 are directly phosphorylated by Src after ischemia (Fig. 2B), and it is consistent with the view from Alavi [23]. While recent findings suggested that ERK signaling participated in hypoxia-induced neurogenesis *in vitro *[35,36], in this study, our data showed that blocking the activation of ERK diminished the ischemia-promoted increase in adult hippocampal progenitor cells of rats (Fig. 3B-a, b), and it further proved that ERK was of great significance in mediating cell proliferation the DG. Taken together, it is convincing to suggest that Src participating in the regeneration of adult hippocampal progenitor cells triggered by ischemia is through mediating the Raf/ERK cascades. CREB is a basic leucine zipper family transcription factor that mediates diverse responses in the nervous system [37]. Our data showed that ischemia also caused continuous activation of CREB in the DG region of hippocampus (Fig. 2C), and inhibition of Src/Raf/ERK pathway by SU6656 and U0126, both of which significantly decreased the p-CREB level (Fig. 2C; Fig. 3C). Meanwhile, there is abundant evidence that CREB is involved in the progress of differentiation and survival, as well as proliferation, of progenitor cells in adult hippocampus [38-40]. More importantly, some recent studies in rats demonstrated that activation of CREB after cerebral ischemia stimulated cell proliferation in the adult DG [28]. Our results indicated that both Src- and ERK- dependent proliferations of adult hippocampal progenitor cells were mediated by activation of CREB, and provided further proof that Src/Raf/ERK cascade was involved in neural cell proliferation evoked by ischemia in DG.

In addition, ischemia insult may also trigger others molecular pathways, which may associate with altering proliferation of progenitor cells. The results (in Fig. 3B) showed there was a distinction between blockage of p - Src and that of p - ERK in the number of DG BrdU-labeling cells, indicating that beside Raf/ERK cascade, there might be some other factors triggered by Src getting involved in this event, such as PI3K/Akt pathway which has also been known to be activated by Src kinase following ischemia/reperfusion in various organs, and plays a pivotal role in cell proliferation, differentiation, and survival [41-43]. On the other hand, one possible mechanism underlying brain ischemia-induced proliferation of neural progenitors is stimulation of tyrosine kinase-coupled receptors by induction of growth factors such as FGF, BDNF and NGF [29]. Brain ischemia-induced cell proliferation is triggered by ERK activation through expression of growth factors and cognate receptors in the DG [29,44]. this report may be explain the phenomenon in our results, CREB phosphorylation is still considerably up-regulated even after SU6656 inhibition compared to the control, and the effects of U0126 upon CREB is much more remarkable. But how these molecular signals concordantly regulate each other, and their contribution in the birth of progenitor cells are unknown, which remain further research.

It is generally accepted that the region of DG is relatively resistant to ischemic injury. Our result also validated that there is no ischemia - induced neuronal death in the DG fields (Fig. 4B-DG). Thus we assume that sustained activation of Src kinase may not interfere with survival of neurons in DG fields after ischemia. However, cerebral ischemia triggers neuronal death in CA1 of hippocampus, and our data suggested Src activation also played a key role in CA1 pyramidal cell death after ischemia (Fig. 4C - CA1). We are therefore confused about which was the key factor in stimulating cell proliferation of DG region, Src kinase or neuronal damage in CA1, as both regions of DG and CA1 were related to activation of Src but with different kinds of neuronal destiny after ischemia. Considering that blockage of activation of ERK did not reduce neuronal death rate of CA1 fields (Fig. 4D - CA1), we suppose that the proliferation of adult progenitor cells in DG is not directly related to injury in the CA1 region evoked by ischemia, and that the different fates of neurons suffering ischemia between DG and CA1 are determined by different signaling pathway-dependent activations of Src kinase. What's more, the Raf/ERK/CREB cascade only mediates cell proliferation of DG but has no association with neuronal death of CA1 after ischemia.

## Conclusion

**Figure 5:**
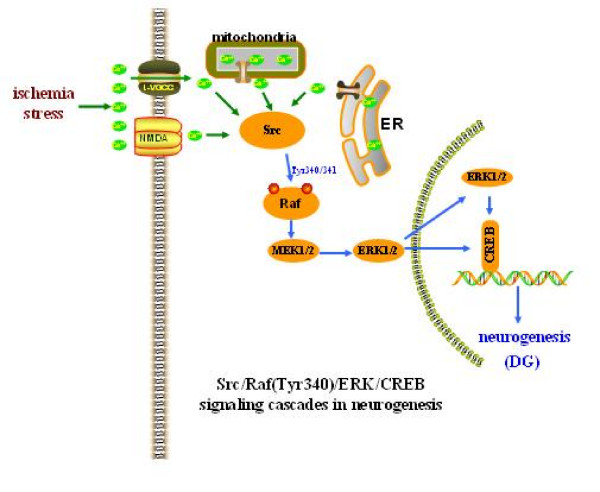
**Suggested pathways through which Src kinase regulates cell proliferation in the DG after ischemia**. Transient global ischemia triggered sustained activation of Src kinase by intracellular Ca^2+ ^influx via NMDA and L-VGCC or released from ER and mitochondria. Subsequently, activated Src induced continuous phosphorylation of ERK through direct phosphorylation of Raf-1 at its Tyr 340/341, and in turn, was translocated from the cytosol to the nucleus where it regulated transcription via CREB and some genes relating to cell proliferation expression.

Following cerebral ischemic insults, the marked stimulation of neuronal cell proliferation by Src mechanisms in the dentate gyrus is partly mediated through the cascade from Ca^2+ ^influx to Raf/ERK activation, and then to CREB phosphorylation, which, in turn, triggers genes related to cell proliferation expression (Fig. 5).

## Methods

### Animals and Induction of ischemia

All animal experiments were carried out in accordance with the Institutional Animal Care and Use Committee and conformed to international guidelines on the ethical use of animals (Guide for the Care and Use of Laboratory Animal, 1996). The animals used in the present study were male Sprague-Dawley rats (Shanghai Experimental Animal Center, Chinese Academy of Science) weighing 250-300 g, maintained in individual cages with a 12-h light/dark cycle (lights on from 08:00-20:00). Transient global cerebral ischemia was induced using four-vessel occlusion as described previously [25]. Rats were anesthetized with intraperitoneal administration of chloral hydrate (300 mg/kg, i.p.). Both vertebral arteries were permanently electrocauterized at the level of the first cervical vertebra within the alar foramina, and a small-diameter silk thread was placed around each carotid artery to facilitate subsequent occlusion. Twenty-four hours after electrocauterization, common bilateral carotid arteries were clamped with microaneurysm clamps for 10 min in animals which were awake and spontaneously ventilated, at which time the clips were removed and the rat was allowed to recover. Rats lost their righting reflex within 30 s and those whose pupils were dilated and unresponsive to light were selected for the experiments. Rectal temperature was maintained at about 37°C during and after ischemia. Sham-operated control rats received an identical procedure without arterial occlusion.

### Drugs and drug treatments

SU6656 (2-oxo-3-(4, 5, 6, 7-tetrahydro-1H-indol-2-ylmethylene)-2,3- dihydro-1H-indole-5-sulfonic acid dimethylamide) is an inhibitor of the Src family tyrosine kinases (Calbiochem, La Jolla, CA, USA). U0126 (1,4-Diamino-2,3-dicyano-1,4-bis(o-aminophenylmercapto)butadiene) is a potent inhibitor of MEK/ERK (Cell Signaling; USA). 1 μl of dimethyl sulfoxide (DMSO) in 4 μl of phosphate-buffered saline (PBS, PH 7.4) as vehicle or SU6656 (100 pmol/animal) or U0126 (1 nmol/animal) was administered using a microinjector into the left cerebral ventricle (from the bregma: anteroposterior, -0.8 mm; lateral, 1.5 mm; depth, -3.5 mm) over 5 min or its vehicle. After the injection, the injector was left in place for an additional 5 min to reduce any possible backflow of the liquid along with the injection void, and occlusion occurred 20 min post the injection. Bromodeoxyuridine (5-bromo-2-deoxyuridine, BrdU), a synthetic nucleoside and an analogue of thymidine commonly used in the detection of proliferating cells in living tissues, was administered by intraperitoneal injections (50 mg/kg body weight at a concentration of 7 mg/ml in sterile saline, Sigma, USA) twice daily during days 5 - 7 after 10 min ischemia or sham operation. The animals were put to death 24 h after the last injection of BrdU.

### Production of protein extracts and Western blotting

For biochemical studies, rats were killed by decapitation at various times post the reperfusion (24 h, 72 h). The hippocampi were quickly removed on ice in a cold room and the hippocampal CA3/DG subfields were dissected on ice as described previously (Guo *et al*., 2006). The separated brain regions were homogenized in 1:10 (w/v) ice-cold homogenization buffer A (HEPES 50, pH 7.4, KCl 100, Na_3_VO_4 _1, NaF 50, PMSF 1 mM) supplemented with 1% mammalian protease inhibitor cocktail (Sigma-Aldrich Co.). Proteins in the cytoplasm and membrane were extracted by centrifugation at 800 × g at 4°C and those in the nucleus were obtained by centrifugation at 14000 × g at 4°C in homogenization buffer B (HEPES 50, pH 7.4, KCl 100, Na_3_VO_4 _1, NaF 50, NP40 1, PMSF 1 mM, 0.5% SDS and 1% cocktail) and then stored at -80°C until assayed. Protein concentrations were determined by a Bradford protein assay [45]. Denatured samples from the extracts (30~40 μg protein) were separated by 10% SDS-polyacrylamide gels and then electrophoretically transferred to nitrocellulose filter membranes (NC, pore size, 0.2 μm, Amersham Biosciences, Piscataway, NJ, USA). The filters were probed with the following antibodies at room temperature for 4 h: rabbit polyclonal anti-Src (1:1000), rabbit monoclonal anti-phospho-Src (Tyr527, 1:1000), mouse polyclonal anti-MAP kinase (1:2000), mouse monoclonal anti-phospho MAP kinase (Thr202/Tyr204, 1:1000), mouse monoclonal anti-phospho-CREB (Ser133, 1:1000), rabbit monoclonal anti-CREB (1:1000) from Cell Signaling Technology (Beverly, MA, USA), rabbit polyclonal anti-phospho-Raf (Tyr340/341, 1:1000) from Calbiochem, mouse monoclonal anti-c-Raf (1:1000) from BD PharMingen (Torrey Pines, CA, USA), and rabbit polyclonal anti-β-actin (1:500) from Boster Biotechnology (WuHan, China). Detection was carried out using horseradish peroxidase (HRP) - conjugated goat anti-rabbit IgG (1:5000, Santa Cruz) and goat anti-mouse IgG (1:5000, ZhongShan Golden Bridge Biotechnology, Beijing, China) and developed via enhanced chemiluminescence (Amersham Biosciences). The optical density of the band in each lane was expressed as fold versus levels from sham operated tissue on the same filter.

### Fixation, Tissue sample preparation and Nissl staining

Rats were deeply anaesthetized with chloral hydrate (300 mg/kg, i.p.) and transcardially perfused with 300 ml saline, followed by 500 ml 4% cold paraformaldehyde in PBS. Brains were removed, post-fixed overnight at 4°C in same solution and then sliced at 40 μm using a vibratome in a bath of physiological saline collected free-floating and stored in 30% ethylene glycol, 30% glycerol, and 0.1 M PBS at - 20°C until processed for immunostaining. Some sections were processed for staining with Toluidine blue (Nissl staining) for histological assessment of damage.

### Immunohistochemistry

For the detection of BrdU immunoreactivity, DNA denaturation was conducted by incubating sections in 50% formamide and 2× sodium citrate solution for 2 h at 65°C, followed by incubation in 2 N HCl for 30 min. Then sections were incubated for 10 min in 0.1 M borate buffer. After being washed in PBS, sections were incubated in 1% H_2_O_2 _for 30 min for the removal of endogenous peroxidases. After several rinses in PBS, sections were incubated in PBS/0.2% Triton X-100/5% goat serum (PBS-TS) for 30 min and then incubated with polyclonal sheep anti-BrdU (Biotin) (1:1000; Abcam, Cambridge, UK) overnight at 4°C. After several rinses in PBS - TS, sections were incubated for 30 min with a streptavidin - HRP complex (1:100; Boster Biotechnology; WuHan, China). BrdU positive (BrdU+) cells were labeled using DAB as chromogen (Zhong Shan -Golden Bridge Biological Technology; Beijing, China). The labeling was imaged with a confocal laser - scanning microscope (Olympus LSM-GB200; Tokyo, Japan) and analyzed with Image-Pro Plus software (Media Cybernetics, Silver Spring, MD, USA).

### Cell counting

To count BrdU - labeled cells after immunohistochemistry, the analysis was conducted using a modified version of the optical fractionator method, on every sixth section in a series of 40 μm coronal sections [46]. All BrdU - labeled cells in the subgranular zone (SGZ) and hilus were counted by an experimenter that was blinded to the code of the sections. BrdU-positive cells regardless of size or shape were visualized and counted through an Olympus BX50 microscope (Olympus, Tokyo, Japan).

To quantitate the survival rate of hippocampal neurons after Nissl staining, cell counts were performed as described previously [47,48]. A rectangular grid housed in one eyepiece was superimposed over 3 medial-lateral sectors (i.e. medial, middle and lateral) of the dorsal CA1 subfield at a level corresponding to the anterior hippocampus (- 1.7 mm posterior to bregma). Only cells with an intact cell membrane, a well-defined nucleus and nucleolus were counted. Using these criteria, the number of normal neurons across all 3 medial - lateral sectors of the CA1 subfield from both hemispheres was summed to yield total CA1 cell counts for each animal.

### Statistical analysis

All data are expressed as mean ± SEM from animals. Statistical significance was assessed by one-way analysis of variance (ANOVA) with the least significant difference test using SPSS software version 10.0. Values of *P *< 0.05 were considered statistically significant.

## Abbreviations

DG: dentate gyrus; BrdU: bromodeoxyuridine; ERK: Extracellular signal - regulated kinase; CREB: cyclic AMP response element-binding protein; MAPK: mitogen-activated protein kinase; SFKs: Src family kinases; PTK: protein tyrosine kinase; SU: SU6656; U0: U0126; 4VO: four-vessel occlusion; i.c.v.: intracerebral ventricular; BSA: bovine serum albumin; PAGE: polyacrylamide gel electrophoresis; Veh: vehicle; FGF: fibroblast growth factor; BDNF: brain-derived neurotrophic factor; NGF: nerve growth factor; ER: endoplasmic reticulum.

## Authors' contributions

HT, BH, JZ carried out the 4-VO model and sample preparation, participated in the western blot analysis, immunohistochemistry, statistical analysis and drafted the manuscript. XH participated in the i.c.v. infusion and cell counting. JG, LL conceived of the study and participated in its design and coordination. All authors read and approved the final manuscript.
